# What Are the Important Factors Influencing the Recruitment and Retention of Doctoral Students in a Public Health Setting? A Discrete Choice Experiment Survey in China

**DOI:** 10.3390/ijerph18189474

**Published:** 2021-09-08

**Authors:** Shimeng Liu, Yingyao Chen, Shunping Li, Ningze Xu, Chengxiang Tang, Yan Wei

**Affiliations:** 1 School of Public Health, Fudan University, Shanghai 200032, China; 19111020032@fudan.edu.cn (S.L.); nzxu17@fudan.edu.cn (N.X.); 14111020034@fudan.edu.cn (Y.W.); 2 NHC Key Laboratory of Health Technology Assessment, Fudan University, Shanghai 200032, China; 3 Centre for Health Management and Policy Research, School of Public Health, Cheeloo College of Medicine, Shandong University, Jinan 250012, China; lishunping@sdu.edu.cn; 4 School of Public Administration, Guangzhou University, Guangzhou 510006, China; tang.chengxiang@gmail.com

**Keywords:** social medicine and health care management, doctoral students, public health, job preference, discrete choice experiment

## Abstract

Objectives: This study aims to investigate the employment preferences of doctoral students majoring in social medicine and health care management (SMHCM), to inform policymakers and future employers on how to address recruitment and retention requirements at CDCs across China. Methods: An online discrete choice experiment (DCE) was conducted to elicit doctoral SMHCM students’ job preferences. The scenarios were described with seven attributes: monthly income, employment location, housing benefits, children’s education opportunities, working environment, career promotion speed, and bianzhi. A conditional logit model and a mixed logit model were used to evaluate the relative importance of the selected attributes. Results: A total of 167 doctoral SMHCM students from 24 universities completed the online survey. All seven attributes were statistically significant with the expected sign and demonstrated the existence of preference heterogeneity. Monthly income and employment location were of most concern for doctoral SMHCM students when deciding their future jobs. Among the presented attributes, working environment was of least concern. For the sub-group analysis, employment located in a first-tier city was more likely to lead to a higher utility value for doctoral students who were women, married, from an urban area, and had a high annual family income. Unsurprisingly, when compared to single students, married students were willing to forgo more for good educational opportunities for their children. Conclusions: Our study suggests that monthly income and employment location were valued most by doctoral SMHCM students when choosing a job. A more effective human resource policy intervention to attract doctoral SMHCM students to work in CDCs, especially CDCs in third-tier cities should consider both the incentives provided by the job characteristics and the background of students. Doctoral students are at the stages of career preparation, so the results of this study would be informative for policymakers and help them to design the recruitment and retention policies for CDCs.

## 1. Introduction

### 1.1. The Importance of SMHCM and the Geographical Imbalance of Health Workforce

The Coronavirus disease 2019 (COVID-19) pandemic placed a spotlight on infectious disease prevention, identification, and population healthcare management [1]. As an interdisciplinary major between medicine, social science, and management science, social medicine and health care management (SMHCM) shoulders an important part of the responsibility of training the public health workforce, who are at the frontline of this and future potential pandemics [2]. Graduates of this major constitute a significant part of the public health workforce in China and are widely employed by various health institutions, including, but not limited to, hospitals, health inspection institutes, centers for disease control and prevention (CDCs), government, academics, and pharmaceutical companies [3,4]. However, a shortage in the public health workforce and its uneven distribution across developed and underdeveloped areas still exist in China’s CDCs [5]. According to a survey conducted by Chinese CDCs in 2020, the density of the public health workforce significantly varied across 31 provinces and had decreased annually, with the aggregate ratio of public health workforce to general population decreasing from 1.47 per 10,000 in 2008 to 1.42 per 10,000 in 2017, which is consistently below the critical shortage threshold of 1.75 per 10,000 recommended by the National Health Commission (NHC) [5]. Despite the importance of SMHCM in public health, it has not been given enough attention by the government and public in China. Evidence worldwide suggests that SMHCM is not as mature a profession as other subjects, such as medicine or nursing; therefore, it needs more financial and policy support in the future [6].

### 1.2. The Necessity for Investigating Doctoral SMHCM Students’ Job Preferences

A high turnover of the public health workforce and a lower willingness to work in the public health system among graduates of related majors contribute significantly to the recruitment and retention problems at CDCs [7]. Recent trends show that many SMHCM graduates have chosen to work elsewhere; for example, in hospitals or pharmaceutical companies [5]. According to the statistics of the NHC, from 2009 to 2018, the amount of health workers at hospitals increased by 58.07%, while the number of personnel at CDCs decreased by 4.5% [8]. To address those problems, there is an urgent need to carefully identify enablers for the implementation of public health functions in the new era, alongside policy implications for an equitable distribution of the public health workforce with a focus on rural or underdeveloped areas, even more so in view of the current pandemic crisis. Both the existing public health workforce and doctoral SMHCM students will be important members of the health workforce in the future. Thus, to better address the recruitment and retention issues and craft corresponding policy interventions, there is a need to further investigate the nature and determinants of doctoral SMHCM students’ job preferences.

### 1.3. Methods for Preferences Elicitation

A variety of approaches has been used to elicit and quantify preferences in a healthcare setting, such as time trade-off (TTO), standard gamble (SG), person trade-off (PTO), and contingent valuation (CV) [9]. Although each of these approaches has merit [10], they are limited in that they are only able to measure preferences according to the trade-offs inferred between two characteristics. There has been growing interest in the application of alternative preference elicitation approaches that are capable of eliciting trade-offs between more than two characteristics; in particular, the discrete choice experiment (DCE). Thus, this study used a DCE survey to elicit the job preferences of doctoral SMHCM students in China. DCE is commonly used, is considered a realistic representation of actual decision-making, and has been shown to be one of the more robust methods to elicit preferences.

### 1.4. Research Progress of DCEs in Students Job Preferences

Evidence suggests that a number of DCE studies have already been conducted for student job preferences in China and many other countries, but not for public health students [11,12,13]. For example, one study conducted in the UK found that medical students value good working conditions significantly more than they value a desirable geographical location [14], while recently published research in China demonstrated that employment location and monthly income were valued most by undergraduate pharmacy students when choosing a job [15]. This study presents the first DCE evidence for doctoral public health students. Because they are completing a PhD, the highest level of education, they have typically already started planning their job career. Hence, wo hope the results of this study can be informative and robust and can assist in more effective human resource policy design for CDCs, especially CDCs in third-tier cities (underdeveloped areas in China).

## 2. Materials and Methods

### 2.1. Sampling

In China, approximately 30 universities offer an SMHCM major [7]. In order to collect as many samples as possible, an anonymous web-based survey was conducted using the Sojump software between 20 October and 12 November 2020. We posted the survey link on WeChat (a popular Chinese social media site) and also sent the link to doctoral SMHCM students at Chinese Universities identified by the authors. These students were asked to circulate the survey links to their classmates and to students they knew in other universities. Based on the simple sampling strategy proposed by Orme [16], the minimum number of respondents required for this study was 83. Considering the possibility of conducting further subgroup analyses, we aimed to enroll a minimum of 150 respondents. Although this sample size is relatively small for a conjoint analysis [17,18], given the limited number of universities that provide SMHCM trainings for PhD students, the number of students who can state their preferences in this research is still reasonable.

### 2.2. Discrete Choice Experiment

DCE is the most common type of ordinal preference method used in health economics and health services research [19]. In DCEs, students are expected to make trade-offs in a series of imperfect job scenarios (each job has advantages and disadvantages) with different attribute profiles. DCEs are grounded in theories [20], which assume that (1) alternatives can be described by their attributes, (2) an individual’s valuation depends upon the levels of these attributes, and (3) choices are based on a latent utility function. The DCE is considered to be a more realistic representation of actual decision-making as it allows for the estimation of overall preferences for any given combination of attributes and is shown to be one of the more robust methods to elicit preferences [21].

### 2.3. Selection of Attributes for the Choice Experiment

We used qualitative research and a literature review to select the attributes to be included in the DCE. From our literature review, an initial set of ten attributes that incorporated personal and employment aspirations (with their levels) were identified, including monthly income, bianzhi, employment location, housing benefits, children’s educational opportunities, working environment, career promotion speed, workload, management style, and training opportunities [22,23,24,25,26]. An iterative qualitative process was undertaken to finesse the attributes and levels. A face-to-face in-depth interview was conducted with seven doctoral SMHCM students from Fudan university and Shandong University, which suggested that the attribute of “management style” and “workload” could be removed as they were not the PhD student’s main concern compared with the other seven attributes when choosing a job. In addition, we consulted two experts in the field of DCE and three experts working in related public health trajectories for the remaining attributes. After the consultation, we retained the attribute of career promotion speed, removed the training and career development opportunity, and adjusted the level of monthly income from CNY 15,000–30,000 to CNY 10,000–25,000, equivalent to USD 1449.1–3622.7 (USD 1 = CNY 6.901 in 2020 based on OECD data). See Table 1 for more details regarding the attributes and levels.

### 2.4. DCE Design

We followed standard approaches for the design of the DCE in order to achieve unbiased, statistical response efficiency [27]. The DCE was based on seven attributes. Three of the seven attributes were described in choice tasks by three response levels, three attributes by two levels, and one attribute by four levels, yielding a total of (e.g., 3 × 3 × 3 × 2 × 2 × 2 × 4) 864 potential combinations. The design approach was informed by Huber and Zwerina [28], the DCE macros for SAS (version 9.4) were used for orthogonal main effect design, and selected profiles were organized into D-efficient choice designs (relative D-Efficiency: 77.9%) [29]. It is common practice in the DCE literature to include only main effects, because it is argued that such effects explain most of the variation in preferences [20]. In such case, only the main effect was estimated in our study. Finally, 36 choice sets were identified and were further divided into three blocks to reduce cognitive burden. Within each version, a single choice set was duplicated to examine the internal consistency of respondent choices. We did not leave respondents an opt-out option. This is consistent with our experiment setting. The doctoral SMHCM students are in the stages of career preparation; nearly all of them will enter the job market after graduation. Moreover, an opt-out may only introduces slight differences into the estimations [30], whereas the forced-choice method leads to more thoughtful responses and better-quality data [31]. All participants were randomized to receive one of the three versions according to their month of birth. (Block 1: January to April; Block 2: May to August; Block 3: September to December). An example of the DCE choice set is provided in Table S1.

### 2.5. Data Collection

In addition to the DCE questions, the online questionnaire also contained questions related to doctoral SMHCM students’ sociodemographic characteristics, job aspirations, occupational planning, and annual family income. A ranking question was conducted prior to the DCE choice sets to further examine the internal predictive validity of the DCE results, in which respondents were asked to rank three attributes (within seven attributes) from most important to least important with respect to their job preferences. At the end of the questionnaire, the respondents were given a task to indicate, on a 5-point scale, the level of difficulty in understanding the 13 DCE choice tasks. The questionnaire was piloted among doctoral SMHCM students at Fudan University and Shandong University, before data collection was conducted between July and October of 2020, to examine the comprehensibility, acceptability, and validity of the questionnaire, with the language and layout being revised thereafter.

### 2.6. Data Analysis

STATA 15.1 was used for all analyses. Descriptive statistics were reported for participants’ socio-demographic characteristics, the ranking results, and the 5-point scale score. The utility (U) associated with a particular job is made up of two components: the deterministic component, $v_{ni}$, and the unobservable component, $\varepsilon_{ni}$. The utility function for the individual, n, associated with job, I, can be specified as:
〖U〗_ni = v_ni + 〖ε〗_ni =〖 β〗_1〖Location〗_(second-tier city) + β_2 〖Location〗_(first-tier city)+ 〖 β〗_3 〖Housing〗_(allowance) + β_4 〖Housing〗_(provided)+ β_5 〖〖Children〗^’ seducation〗_(good) + β_6 〖Promotion〗_(3 year)+ β_7 〖Promotion〗_(1 year) + β_8 〖Working environment 〗_(better)+ 〖 β〗_9 〖bianzhi 〗_offer + β_10 Monthly Income+〖 ε〗_ni(1)


Two econometric models were considered: the conditional logit (Clogit) and the mixed logit (MIXL), which uses random coefficients to accommodate potential unobserved preference heterogeneity [32]. The Akaike information criterion (AIC) and Bayesian information criterion (BIC) were used for model comparisons [33,34]. The sensitivity of the final model was tested by allowing for 500, 1000, 1500, and 2000 Halton draws, showing no significant effect on parameters [35]. The final model used 500 draws.

Attributes were coded to dummy variables. When estimating MIXL, all coefficients were specified as random (normally distributed), except for monthly income, which was fixed to facilitate a calculation of willingness to pay (WTP; that is, the relative monetary value that doctoral SMHCM students place on different aspect of the job attribute levels: (−(β(1,2…9))/β10, where β_10 is the salary coefficient and β_((1,2…9)) is the coefficient for attribute level 1, 2…9). Finally, we also conducted an uptake rate study to understand to what extent the probability of choosing a given post changes as the levels of the attributes are changed.

## 3. Results

### 3.1. Respondents

A total of 193 individuals from 41 universities participated in the online survey, among which 26 (from 17 universities) were excluded because their universities did not have an SMHCM major; we therefore took them as invalid data. Among the remaining 167 participants (24 universities from 13 provinces), only 14 (8.4%) participants failed the internal consistency test (internal predictive validity), suggesting a very high level of engagement among the participants. The analysis sample (*n* = 153) had a mean age of 28.8 years (SD = 4.5). Most were female (62.1%), came from urban areas (65.4%), and were single (69.9%). Around 79.1% of the PhD students had decided to do a major-related job after graduation, while 18.9% has not made up their minds. See Table 2 for more details. For the ‘5-point scale’ question, 61 respondents (39.9%) thought it was easy or very easy to understand the 13 DCE questions, 66 respondents (43.1%) thought it was normal, and only 26 respondents (17.0%) thought it was difficult or very difficult, suggesting a high data quality of DCEs in our survey.

### 3.2. DCE Results

The DCE results reported were all based on the analysis sample (3672 observations from 153 doctoral SMHCM students). A sensitivity analysis was undertaken, including the 14 participants who failed the internal consistency test (Table S2), and these changes did not materially affect the findings. The AIC and BIC values suggested that the MIXL estimates were preferable to the Clogit estimates for the analysis sample and the results from MIXL were not substantially different from the Clogit. As such, the main paper reports the MIXL estimates (Table 3), and the Clogit estimates are presented in Table S3.

Statistical significance of all the mean preference parameters suggest that the selected attributes are all significant predictors of the job choice. Some estimated standard deviations are significant, indicating the existence of preference heterogeneity. Results from the MIXL show that doctoral SMHCM students strongly favored first-tier cities over third-tier cities (β = 1.576; *p* < 0.001). Doctoral SMHCM students also exhibited strong preferences for provided housing compared with no housing benefits (β = 1.004; *p* < 0.001), as well as bianzhi compared with no bianzhi (β = 0.964; *p* < 0.001). Doctoral SMHCM students expressed a preference for 1 year to get promoted (β = 0.633; *p* < 0.001), as well as good children’s education (β = 0.498; *p* < 0.001). Better working environment was deemed the least important (β = 0.344; *p* < 0.001).

### 3.3. Willingness to Pay

**Figure 1:**
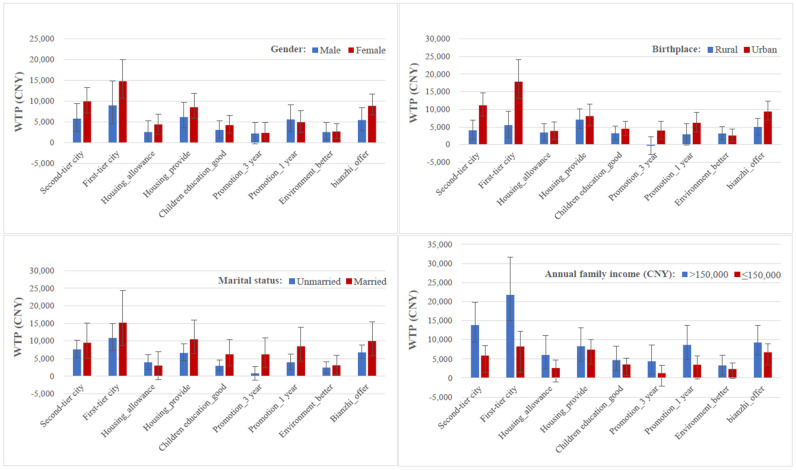
Willingness to pay estimation for subgroup population.

The WTP analysis revealed that doctoral SMHCM students were willing to forgo CNY 12,409.4 to attend a job in a first-tier city rather than in a third-tier city. Doctoral SMHCM students were willing to forgo CNY 7905.5 for housing provided rather than no housing benefits provide. In terms of bianzhi, they were willing to forgo CNY 7590.6 to get a job with bianzhi. The results of selective sub-group analyses are presented in Table 4 and Figure 1. For the subgroup analysis, a job in a first-tier city was more likely to lead to a higher utility value for doctoral SMHCM students who were women, married, coming from an urban area, and had a high annual family income. In addition, compared with female students, the male students were willing to forgo more for a job with 1 year to get promoted.

### 3.4. Uptake Rate

**Figure 2:**
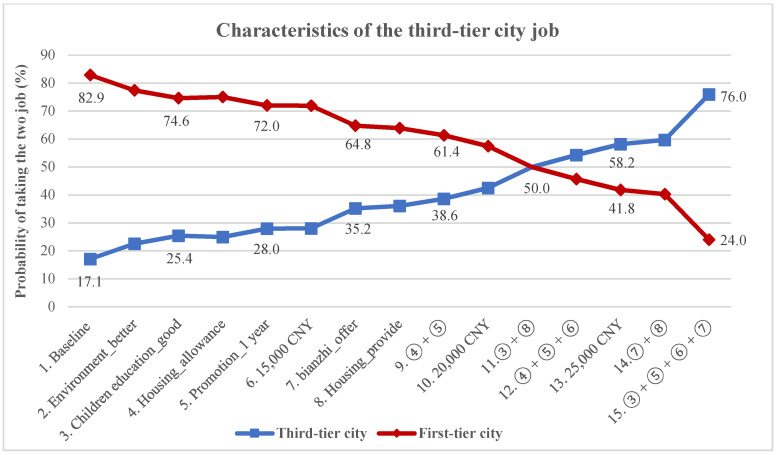
Simulated preferences for job posting under different potential policy scenarios.

The uptake rate results are shown in Figure 2. The initial (baseline: CNY 10,000 monthly income; no housing benefits; ordinary children’s education opportunities; career promotion after 5 year; no bianzhi, ordinary working environment) probability of taking a third-tier city job is 17.1%, hence the probability of taking a first-tier city job is 82.9%. For the single incentives, only increasing monthly income from CNY 10,000 to 25,000 made the probability of choosing a third-tier city job (58.2%) exceed the probability of choosing a job in a first-tier city (41.8%). For the given multiple incentives, the policy “③ + ⑤ + ⑥ + ⑦” was the most attractive one, as it can increase the probability of taking a third-tier city job to 76.0%.

## 4. Discussion

Our study shows that the majority of the PhD students prefer to find a job in the university or research institution; only a few students plan to work at CDCs. Although respect for the CDC workforce was significantly enhanced after the COVID-19 outbreak, their wages, however, still decreased [36]. Turnover is a common phenomenon, both in national CDCs and local CDCs in China because of the low salary [7]. Our study confirms that financial incentives are still the most important lever for recruitment and retention, and when compared across employment locations, the magnitude of the incentive has an effect. For example, a CNY 5000 (USD 724.5) salary increase from baseline was relatively ineffective but became significant when further increased (Figure 2).

Among non-monetary attributes, working in first-tier cities is the most important factor, especially for students from urban areas. The results of ranking job posting attributes according to their importance in Figure S1 again confirmed the importance of employment location. Large metropolitan centers offer more career and educational advancement, better employment prospects, and easier access to lifestyle-related services and amenities [37]. Studies from other countries have reported that the more urban the job, the more it will be preferred [38,39]. In addition, students from urban areas showed a much stronger preference to work in a first-tier city. Therefore, as one possible emergent option, attracting and retaining doctoral SMHCM students from a rural background for the grassroots CDCs might be more effective.

In teams of housing benefits, providing housing allowance is moderately effective, but providing housing is a very powerful non-financial strategy. This shows the importance of providing housing for doctoral SMHCM students when choosing a job. Other studies have also shown similar results [40]. In recent years, although the Chinese government has always adhered to the policy that ‘houses are used for living, not for speculation’, and local governments have also implemented a series of measures, such as restricting the purchase and loan of houses and increasing the supply of affordable housing, housing prices still exceed the affordability of ordinary office workers [41]. Constrained by their financial capacity, the CDCs in third-tier cities may not be able to provide housing for their employees, but housing benefits, coupled with other incentives such as good educational resources, may work equally well. Other research also found that a bundle of incentives, such as housing combined with education opportunities or an improved working environment, are more likely to be effective in retaining health workers in the long term [42].

Contrary to our previous studies with heath administration [22], nurse [23], or medical students [43], which found that bianzhi has the lowest utility in job preferences, bianzhi is another important non-monetary factor that influenced the doctoral SMHCM students job choice in this study. In China, bianzhi refers to the authorized number of personnel (the number of established posts) in a party or government administrative organ, a service organization, or a working unit; a job with bianzhi means more stability [44]. This is perhaps because the respondents in this study were older, with an average age of 28.8, and some of them had started a family, so a job with bianzhi may have been more important for them. This suggests that, to avoid brain-drain from the CDC system, it is necessary to prepare positions with bianzhi for the more important roles, and the quantity of bianzhi allocated for high-level public health talents may increase based on needs.

Career promotion speed is another important nonmonetary factor, especially for male doctoral SMHCM students. Similar results have been obtained in other human resource DCE-based studies in low- and middle-income countries [40,45]. Snow et al. [46] indicated that the absence of senior posts in underdeveloped areas is an important factor associated with the feeling of “professional imprisonment” identified by those working in rural and remote posts. Another study conducted in China found that the most important factor influencing job satisfaction in CDCs was personal development [47]. In this case, developing clear career paths for rural and remote area posts and adopting strategies to increase public recognition are strongly recommended strategies.

The children’s education opportunities attribute was found to have a relatively smaller effect on doctoral SMHCM student’s job preferences than employment location, housing benefits, or career promotion speed. It seems contrary to the study conducted in Nepal [48] in which children’ education was found to be a much stronger predictor of choice. It could be that most of the doctoral SMHCM students we studied had not started a family, so perhaps their future children’s education was not among their main concerns. The subgroup analysis in our study also strengthened the above assumption that married doctoral students have a stronger preference for children’s education compared with unmarried doctoral students.

Working environment was the least important factor for doctoral SMHCM student’s job preferences. It was contrary to our previous studies, which strongly suggested a preference for improved working environments [22]. This finding is consistent with the results of an earlier quantitative study in which working environment was not thought of as a major contributing factor towards job choice for the doctoral students in China [49]. This suggests that changing the working environment may not be an effective or optimal method to improve recruitment and retention problems for China’s CDCs.

The pandemic of COVID-19 highlights the importance of strengthening public health systems. In the future, the demand for a public health workforce in disease control systems will increase. In addition to our study, other studies have also found that many public health graduates were unwilling to devote themselves to CDCs [5,50]. To address the potential challenge of a human resources shortage in the disease control system of China, further qualitative research, such as in-depth interviews and focus group discussions involving doctoral SMHCM students, is required to determine the specific reasons why they are unwilling to work at CDCs.

There are several limitations in this study. First, the generalizability of the study findings may be limited by the convenience sampling approach. It is not possible to identify the statistics of the target population of SMHCM PhD students currently being trained at universities in China, so the representativeness of our sample could not be fully assessed. It should also be noted that, while 153 students seems to be a relatively small sample, each had responded to 12 choice questions, resulting in a total sample size of 3672 choice observations for data analysis. The mean preference coefficients (as shown in Table 3) were mostly highly significant. Second, DCE analysis stems from the fact that a choice experiment does not offer a multitude of attributes because the choice task becomes difficult and respondents are less willing to critically appraise each attribute as the list grows. Not all potentially important attributes, such as workload, were assessed. Third, the data collected in the DCEs were based on choices among hypothetical job alternatives, and differences may arise between students’ stated and actual choices. Finally, the respondents in this study were not limited to final year doctoral SMHCM students. Though job preferences may vary between PhD students at different grades, given the limited sample size, we were unable to examine this difference.

## 5. Conclusions

Although China has conducted a series of DCE-based studies on graduates, the respondents were mainly undergraduate graduates [22,23,43,51]. To the best of our knowledge, this is the first study using DCE methodology to investigate the job preferences of public health related major’s doctoral students internationally. Our study suggests that monthly income and employment location were the most important attributes that impact the student’s job choices. A more effective human resource policy intervention to attract doctoral SMHCM students to work in CDCs, especially CDCs in third-tier cities, should consider both the incentives of the job itself and the background of students. Doctoral SMHCM students are in the stages of career preparation, so the results of this study will be more effective to inform policymakers regarding the design of recruitment and retention policies in the public health setting.

## Figures and Tables

**Table 1:** Attributes and attribute levels.

Attribute	Level	Description
Monthly income	CNY 10,000	Pre-tax salary
	CNY 15,000	
	CNY 20,000	
	CNY 25,000	
Employment location	First-tier city	Represents the larger cities, such as Beijing, Shanghai, Shenzhen, and Guangzhou
	Second-tier city	Represents the medium-sized cities, such as Qingdao and Xiamen
	Third-tier city	Represents the minor cities, such as Weifang and Luoyang
Housing benefits	No housing benefits	Housing provided means a decent house is provided.
	Housing allowance provided	
	Housing provided	
Children’ education opportunities	Ordinary	The educational opportunities available for children (including elementary school, middle school, or high school) in the workplace.
	Good	
Career promotion speed	1 year later	The number of years you would have to work before being eligible for promotion.
	3 year later	
	5 year later	
Working environment	Ordinary	Refers to the physical and social environment associated with the work
	Better	
bianzhi	None	A job with bianzhi means more stability
	Offer	

US$1 = CNY 6.901; https://data.oecd.org/conversion/exchange-rates.htm (2020). (accessed on 18 February 2021).

**Table 2:** Respondent characteristics.

	Full Sample: *n* = 167		Analysis Sample: *n* = 153		Excluded Sample: *n* = 14		χ^2^ (*p*-Value)
	*n*	%	*n*	%	*n*	%	
Age (year), Mean ± SD	28.8	4.5	28.8	4.5	29.1	3.5	
Gender							0.026 (0.871)
Male	63	37.7	58	37.9	5	35.7	
Female	104	62.3	95	62.1	9	64.3	
Birthplace							
Rural	59	35.3	53	34.6	6	42.9	
Urban	108	64.7	100	65.4	8	57.1	
Marital status							0.529 (0.912)
Unmarried	118	70.7	107	69.9	11	78.6	
Married	48	28.7	45	29.4	3	21.4	
Divorced/Widow	1	0.6	1	0.7	0	0	
Monthly consumption (CNY)							3.465 (0.629)
<1500	33	19.8	29	19.0	4	28.6	
1500–2500	70	41.9	65	42.5	5	35.7	
2500–3500	23	13.8	21	13.7	2	14.3	
3500–4500	14	8.4	14	9.2	0	0	
4500–5500	4	2.4	3	2.0	1	7.1	
>5500	23	13.8	21	13.7	2	14.3	
Annual family income (CNY)							2.865 (0.826)
<50,000	29	17.3	25	16.3	4	28.6	
50,000–100,000	39	23.3	36	23.5	3	21.4	
100,000–150,000	37	22.2	33	21.6	4	28.6	
150,000–200,000	22	13.2	21	13.7	1	7.1	
200,000–250,000	12	7.2	11	7.2	1	7.1	
250,000–300,000	8	4.8	8	5.2	0	0	
>300,000	20	12.0	19	12.4	1	7.1	
Will you take a job related to your major after graduation?							0.971 (0.615)
Yes	131	78.4	121	79.1	10	71.4	
No	3	1.8	3	2.0	0	0	
Not sure	33	19.8	29	18.9	4	28.6	
Career planning (multiple-choice: Times was selected)							
University or scientific research institution			126				
Hospital			63				
CDCs			18				
Government agency			71				
Pharmaceutical company			39				
Others			6				

SD: standard deviation; CNY: Chinese yuan; CDC: Centers for Disease Control and Prevention.

**Table 3:** MIXL estimates and WTP (*n* = 153).

Attributes and Levels	β	SE	SD	SE	WTP (CNY)	95% CI	
Employment location (ref: Third-tier city)							
Second-tier city	1.080 ***	0.147	0.931 ***	0.186	8503.9	6424.4	10,799.6
First-tier city	1.576 ***	0. 220	2.045 ***	0.238	12,409.4	9184.9	16,177.9
Housing benefits (ref: No housing benefits)							
Housing allowance provided	0.480 ***	0.119	0.015	0.183	3779.5	1984.0	5600.5
Housing provided	1.004 ***	0.138	0.547 ***	0.177	7905.5	5910.8	10,194.6
Children’s education opportunities (ref: Ordinary)							
Good	0.498 ***	0.090	0.398 ***	0.152	3921.3	2531.4	5437.9
Career promotion speed (ref: 5 year)							
3 year	0.287 ***	0.112	0.004	0.196	2259.8	526.4	4076.3
1 year	0.633 ***	0.124	0.609 ***	0.197	4984.3	3083.8	7047.3
Working environment (ref: Ordinary)							
Better	0.344 ***	0.082	0.188	0.292	2708.7	1467.3	4007.9
bianzhi (ref: None)							
Offer	0.964 ***	0.115	0.732 ***	0.126	7590.6	5890.9	9475.5
Monthly income	0.000127 ***	0.000011					
LR chi2(10)	161.950						
Number of observations	3672						
Log likelihood	−914.985						
AIC	1867.971						
BIC	1985.932						

*** *p* < 0.01; _β: coefficient; WTP: willingness to pay; CNY: Chinese yuan; SD: standard deviation; SE: standard error; 95% CI: 95% confidence intervals; AIC: Akaike information criterion; BIC: Bayesian Information Criterion.

**Table 4:** Subgroup analyses.

Attributes and Levels	Male (*n* = 58)				Female (*n* = 95)			
	β	SE	SD	SE	β	SE	SD	SE
Second-tier city	0.803 ***	0.235	0.912 ***	0.288	1.331 ***	0.211	1.102 ***	0.279
First-tier city	1.248 ***	0.364	2.276 ***	0.462	1.979 ***	0.306	2.107 ***	0.338
Housing allowance provided	0.357 *	0.188	0.010	0.243	0.593 ***	0.171	0.102	0.571
Housing provided	0.860 ***	0.198	0.139	0.486	1.145 ***	0.203	0.846 ***	0.231
Good children’s education opportunities	0.428 ***	0.133	0.043	0.356	0.572 ***	0.130	0.612 ***	0.192
Career Promotion: 3 year	0.307 *	0.183	0.047	0.307	0.316 **	0.153	0.031	0.232
Career Promotion: 1 year	0.779 ***	0.219	0.642 **	0.282	0.657 ***	0.173	0.662 ***	0.245
Working environment: better	0.357 **	0.139	0.254	0.318	0.368 ***	0.121	0.399 **	0.190
bianzhi: offer	0.759 ***	0.180	0.819 ***	0.229	1.189 ***	0.165	0.755 ***	0.178
Monthly income	0.000139 ***	0.000020			0.000133 ***	0.000017		
Attributes and levels	Unmarried (*n* = 107)				Married (*n* = 45)			
	β	SE	SD	SE	β	SE	SD	SE
Second-tier city	1.011 ***	0.171	0.872 ***	0.226	1.298 ***	0.336	1.382 ***	0.416
First-tier city	1.435 ***	0.249	1.960 ***	0.302	2.076 ***	0.500	2.756 ***	0.618
Housing allowance provided	0.527 ***	0.142	0.003	0.208	0.417	0.263	0.348	0.531
Housing provided	0.878 ***	0.155	0.533 **	0.216	1.434 ***	0.325	0.403	0.408
Good children’s education opportunities	0.395 ***	0.097	0.223	0.298	0.855 ***	0.233	0.659 **	0.262
Career Promotion: 3 year	0.108	0.128	0.014	0.210	0.847 ***	0.271	0.100	0.416
Career Promotion: 1 year	0.527 ***	0.145	0.626 ***	0.226	1.156 ***	0.302	0.759 **	0.379
Working environment: better	0.324 ***	0.104	0.442 **	0.175	0.428 **	0.181	0.030	0.317
bianzhi: offer	0.901 ***	0.124	0.555 ***	0.158	1.362 **	0.327	1.224 ***	0.295
Monthly income	0.000133 ***	0.000014			0.000137 ***	0.000025		
Attributes and levels	Rural (*n* = 53)				Urban (*n* = 100)			
	β	SE	SD	SE	β	SE	SD	SE
Second-tier city	0.586 ***	0.211	0.676 **	0.322	1.367 ***	0.205	1.111 ***	0.254
First-tier city	0.801 ***	0.269	1.341 ***	0.281	2.194 ***	0.330	2.405 ***	0.342
Housing allowance provided	0.496 ***	0.187	0.070	0.311	0.474 ***	0.158	0.067	0.280
Housing provided	1.031 ***	0.210	0.333	0.408	0.997 ***	0.180	0.662 ***	0.222
Good children’s education opportunities	0.464 ***	0.140	0.260	0.311	0.548 ***	0.118	0.483 **	0.208
Career Promotion: 3 year	−0.051	0.178	0.013	0.271	0.486 ***	0.149	0.028	0.258
Career Promotion: 1 year	0.421 *	0.221	0.909 ***	0.253	0.753 ***	0.161	0.469 *	0.265
Working environment: better	0.452 ***	0.137	0.299	0.302	0.313 ***	0.107	0.110	0.369
bianzhi: offer	0.723 ***	0.177	0.759 ***	0.213	1.145 ***	0.159	0.760 ***	0.179
Monthly income	0.000145 ***	0.000020			0.000123 ***	0.000015		
Attributes and levels	≤150,000 CNY (*n* = 94)				>150,000 CNY (*n* = 59)			
	β	SE	SD	SE	β	SE	SD	SE
Second-tier city	0.834 ***	0.178	0.940 ***	0.258	1.523 ***	0.277	1.050 ***	0.291
First-tier city	1.169 ***	0.272	2.035 ***	0.316	2.396 ***	0.399	2.172 ***	0.439
Housing allowance provided	0.371 **	0.153	0.016	0.237	0.668 ***	0.207	0.111	0.336
Housing provided	1.057 ***	0.171	0.397	0.280	0.912 ***	0.234	0.610 *	0.280
Good children’s education opportunities	0.504 ***	0.112	0.319	0.228	0.519 ***	0.158	0.639 ***	0.234
Career promotion speed: 3 year	0.184	0.144	0.045	0.219	0.487 **	0.192	0.006	0.390
Career Promotion speed: 1 year	0.502 ***	0.159	0.614 **	0.238	0.953 ***	0.221	0.585 *	0.311
Working environment: better	0.341 ***	0.110	0.311	0.278	0.364 **	0.141	0.157	0.301
bianzhi: offer	0.958 ***	0.157	0.851 ***	0.166	1.022 ***	0.185	0.601 ***	0.224
Monthly income	0.000142 ***	0.000002			0.000110 ***	0.000018		

* *p* < 0.10; ** *p* < 0.05; *** *p* < 0.01; β: coefficient; SD: standard deviation; SE: standard error.

## Data Availability

The data used and/or analyzed during the study are available from the corresponding author on reasonable request.

## Supplementary Materials

The following are available online at https://www.mdpi.com/article/10.3390/ijerph18189474/s1, Figure S1: Numbers of job attributes chosen as determining factors with different levels, Table S1: Example combination of choice: Which of these jobs would you prefer?, Table S2: Mixed logit estimates (*n* = 167), Table S3: Conditional logit estimates (*n* = 153).

## Abbreviations

DCE	Discrete Choice Experiment
WTP	Willingness to Pay
SMHCM	Social Medicine and Health Care Management
CDC	Centers for Disease Control and Prevention
CNY	Chinese Yuan
95% CI	95% Confidence Intervals
AIC	Akaike Information Criterion
BIC	Bayesian Information Criterion
SE	Standard Error
SD	Standard Deviation
