# Supplementary material for: What Are the Important Factors Influencing the Recruitment and Retention of Doctoral Students in a Public Health Setting? A Discrete Choice Experiment Survey in China
Source: Int J Environ Res Public Health. 2021 Sep 8;18(18):9474. doi: 10.3390/ijerph18189474 (PMC8467983; doi:10.3390/ijerph18189474)
Supplement: Supplementary file 1 [file ijerph-18-09474-s001.zip › Table S1.pdf]

Table S1 Example combination of choice: Which of these jobs would you prefer?

Attributes/levels	Job 1	Job 2
Monthly income	25,000 CNY	10,000 CNY
Employment location	Second-tier city	First-tier city
Housing benefits	House provided	No housing benefits
Children' education opportunities	Ordinary	Good
Career promotion speed	3 year later	1 year later
Working environment	Ordinary	Better
bianzhi	Offer	None
**Which of these jobs would you prefer?**		
