# Supplementary material for: What Are the Important Factors Influencing the Recruitment and Retention of Doctoral Students in a Public Health Setting? A Discrete Choice Experiment Survey in China
Source: Int J Environ Res Public Health. 2021 Sep 8;18(18):9474. doi: 10.3390/ijerph18189474 (PMC8467983; doi:10.3390/ijerph18189474)
Supplement: Supplementary file 1 [file ijerph-18-09474-s001.zip › Table S2.pdf]

Table S2 Mixed logit estimates (n=167)

Attributes and levels	β	SE	SD	SE
Employment location (ref: Third-tier city)				
Second-tier city	1.033***	0.138	0.917***	0.177
First-tier city	1.544***	0.197	1.822***	0.216
Housing benefits (ref: No housing benefits)				
Housing allowance provided	0.406***	0.110	0.100	0.249
Housing provided	0.945***	0.124	0.411**	0.206
Children' education opportunities (ref: Ordinary)				
Good	0.451***	0.081	0.317**	0.157
Career promotion speed (ref: 5 year)				
3 year	0.306***	0.105	0.017	0.179
1 year	0.659***	0.120	0.658***	0.185
Working environment (ref: Ordinary)				
Better	0.334***	0.080	0.319**	0.162
bianzhi (ref: None)				
Offer	0.870***	0.106	0.735***	0.120
Monthly income	0.000122***	0.000		
LR chi2(9)	152.27			
Number of observations	4008			
Log likelihood	-1028.72			

** $p < 0.05$; *** $p < 0.01$; β, coefficient; SD, standard deviation; SE, standard error.
