# Supplementary material for: What Are the Important Factors Influencing the Recruitment and Retention of Doctoral Students in a Public Health Setting? A Discrete Choice Experiment Survey in China
Source: Int J Environ Res Public Health. 2021 Sep 8;18(18):9474. doi: 10.3390/ijerph18189474 (PMC8467983; doi:10.3390/ijerph18189474)
Supplement: Supplementary file 1 [file ijerph-18-09474-s001.zip › Table S3.pdf]

Table S3 Conditional logit estimates (n=153)

Attributes and levels	β	SE
Employment location (ref: Third-tier city)		
Second-tier city	0.766***	0.102
First-tier city	1.040***	0.140
Housing benefits (ref: No housing benefits)		
Housing allowance provided	0.372***	0.075
Housing provided	0.668***	0.085
Children' education opportunities (ref: Ordinary)		
Good	0.317***	0.057
Career promotion speed (ref: 5 year)		
3 year	0.146*	0.077
1 year	0.386***	0.093
Working environment (ref: Ordinary)		
Better	0.216***	0.055
bianzhi (ref: None)		
Offer	0.599***	0.066
Monthly income	0. 000087***	7.37e-06
Wald chi2(10)	273.42	
Number of obs	3672	
Log likelihood	-995.96	
AIC	2011.923	
BIC	2074.008	

* $p < 0.10$; *** $p < 0.01$; β, coefficient; SE, standard error; AIC, Akaike information criterion; BIC, Bayesian Information Criterion.
