# Supplementary figures and images for: What Are the Important Factors Influencing the Recruitment and Retention of Doctoral Students in a Public Health Setting? A Discrete Choice Experiment Survey in China
Source: Int J Environ Res Public Health. 2021 Sep 8;18(18):9474. doi: 10.3390/ijerph18189474 (PMC8467983; doi:10.3390/ijerph18189474)

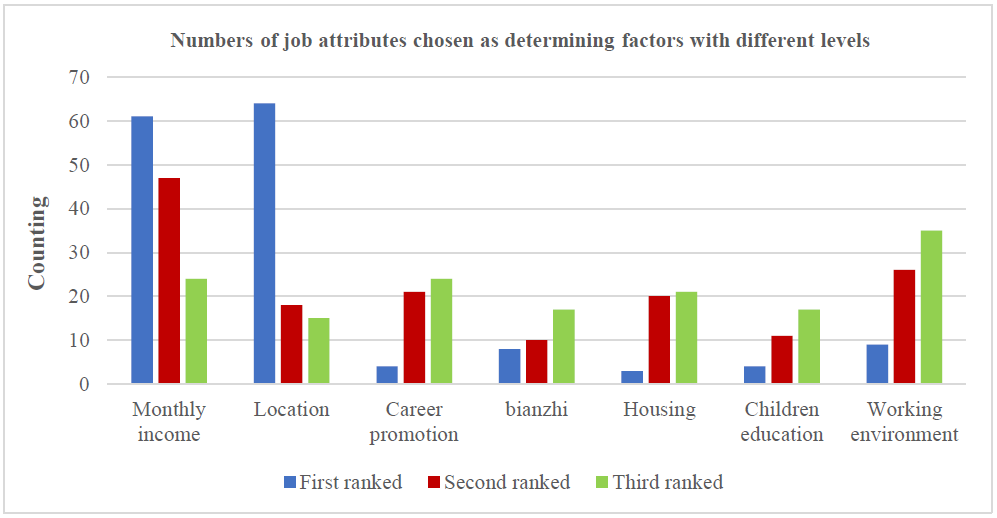

Supplement: Supplementary file 1 [file ijerph-18-09474-s001.zip › Figure S1.png]
